# Taxonomic, Phylogenomic and Bioactivity Profiling of Novel Phycosphere Bacterium from Model Cyanobacterium *Synechococcus elongatus* PCC 7942

**DOI:** 10.3390/md22010036

**Published:** 2024-01-07

**Authors:** Xiaoling Zhang, Jiaquan Xu, Jun Dai, Lei Zhang, Lijuan Feng, Xiaoqing Tian, Qiao Yang

**Affiliations:** 1 Department of Marine Chemistry, College of Marine Science and Technology, Zhejiang Ocean University, Zhoushan 316022, China; 2 Cooperative Innovation Center of Industrial Fermentation (Ministry of Education & Hubei Province), Key Laboratory of Fermentation Engineering (Ministry of Education), National “111” Center for Cellular Regulation and Molecular Pharmaceutics, College of Bioengineering, Hubei Key Laboratory of Industrial Microbiology, Hubei University of Technology, Wuhan 430068, China; 3 ABI Group, Laboratory of Phycosphere Microbiology, Zhejiang Ocean University, Zhoushan 316021, China; 4 Zhejiang Provincial Key Laboratory of Petrochemical Pollution Control, Zhejiang Ocean University, Zhoushan 316022, China; 5 Donghai Laboratory, Zhoushan 316022, China; 6 East China Sea Fisheries Research Institute, Chinese Academy of Fishery Sciences, Shanghai 200090, China; 7 State Key Laboratory of Swine and Poultry Breeding Industry, Institute of Animal Science, Guangdong Academy of Agricultural Sciences, Guangzhou 510640, China

**Keywords:** phycosphere microbiota, algae–bacteria interactions, marine model cyanobacterium, bioactive exopolysaccharides, microalgae growth-promoting bacterium, CO_2_ fixation efficiency by microalgae, *Synechococcus elongatus* PCC 7942

## Abstract

Phycosphere niches host rich microbial consortia that harbor dynamic algae–bacteria interactions with fundamental significance in varied natural ecosystems. Hence, culturing the uncultured microbial majority of the phycosphere microbiota is vital for deep understanding of the intricate mechanisms governing the dynamic interactions, and also to provide novel and rich microbial resources, and to discover new natural bioactive metabolites. *Synechococcus elongatus* PCC 7942 is a robust model cyanobacterium widely used in environment, synthesis biology, and biotechnology research. To expand the number of novel phycosphere species that were brought into culture and to discover the natural bioactivities, we presented a new yellow-pigmented bacterium named ABI-127-1, which was recovered from the phycosphere of PCC 7942, using an optimized bacterial isolation procedure. Combined polyphasic taxonomic and phylogenomic characterization was performed to confidently identify the new isolate as a potential novel species belonging to the genus *Qipengyuania*. The observed bioactivity of strain ABI-127-1 with promoting potential towards the growth and CO_2_ fixation efficiency of the host microalgae was measured. Additionally, the bacterial production of active bioflocculant exopolysaccharides was evaluated after culture optimization. Thus, these findings revealed the potential environmental and biotechnological implications of this new microalgae growth-promoting bacterium isolated from the phycosphere microenvironment.

## 1. Introduction

Phytoplankton are key primary producers in the ocean and freshwater ecosystems [1,2]. Among them, *Synechococcus* is one of the unicellular photosynthetic cyanobacteria with wild distribution, which is responsible for a large part of global oceanic primary production and carbon cycling [3,4]. Different cyanobacterial components (e.g., pigments, proteins, polysaccharides, and lipids) display varied biological availability during the decomposition process. However, the majority of *Synechococcus*-derived organic matter is easily labile for use by the heterotrophic bacterial community [5]. Thus, through varied interactions, marine phytoplanktons and their heterotrophic bacterial communities compose a closely related regulator alliance in the oceans by acting as the dominant primary producers (phytoplankton) and biogeochemical cycle drivers (bacteria), respectively [6]. The kinds of algae–bacteria interactions (ABI) usually span mutualism, commensalism, antagonism, parasitism, and competition, and thus underpin most functions of marine ecological processes [7]. Thus, the phycosphere niches host rich microbial consortium, and thus are regarded as the boundary of phytoplankton holobionts and the ecological interface for complex and dynamic algae–bacteria interactions [8]. Presently, it is becoming increasingly clear that these complex associations usually exert profound influences on fundamental processes including primary production, biogeochemical cycles, phycotoxins biosynthesis, and microbial loops in the oceans [9]. Within this microscopic interface, exopolysaccharides or extracellular polysaccharides (EPS) secreted by both interactive sides are regarded as one vital component of the biological matrix, which embeds the proliferating cells and promotes colony formation during algae–bacteria interactions [10,11,12]. 

Previous studies have confirmed that the family *Erythrobacteraceae* in the order *Sphingomonadales* within the class *Alphaproteobacteria* is one of the predominant bacterial communities among various phycosphere microbiota found in nature [13,14,15]. Among the 19 currently validated members of *Erythrobacteraceae*, the genus *Qipengyuania* was first described in 2015, with the species type *Qipengyuania sediminis* [16]. Currently, the genera comprises 31 valid type species (https://lpsn.dsmz.de/genus/qipengyuania, accessed on 6 June 2023) isolated from various habitats [17,18,19]. It may indicate that this genus may be widely distributed in coastal environments like the *Roseobacter* lineage [20]. In recent years, members of *Qipengyuania* have attracted increasing attention due to their diverse potential applications in agriculture, biotechnology, and industry [21]. 

Still, only a minority of marine microorganisms can be cultured in the laboratory and the enormous bioprospecting potential of uncultured diversity remains unexplored [22]. To improve our understanding of uncultured microbial diversity, it is essential to increase our capacity for bringing microorganisms from the environment into culture [23,24,25]. During the execution of our Phycosphere Microbiome Project (PMP) conveying the microbial diversity of various phycosphere microbiota recovered from the ocean and freshwater ecosystems [26,27,28,29,30], a bioactive yellow-pigmented bacterium named ABI-127-1 was isolated from the phycosphere of *Synechococcus elongatus* PCC 7942, which is a versatile and robust model cyanobacterial strain widely used in the environment, such as in carbon fixation and blue carbon, capturing and acclimation, synthesis biology, and biotechnology research such as green biofuel production [31,32,33,34]. In this study, we proposed a new isolate, strain ABI-127-1, to represent a potential novel species of the genus *Qipengyuania* within the family *Erythrobacteraceae* based on combined polyphasic taxonomic and phylogenomic characterizations. Moreover, various bioactivities of strain ABI-127-1 either acting as new microalgae-growth-promoting bacterium (MGPB), or the bacterial bioflocculanting EPS, as well as the bacterial enhanced carbon fixation efficiency via microalgae, were also measured. 

## 2. Results and Discussion

### 2.1. Morphological and Biochemical Characterization of Strain ABI-127-1

**Figure 1:**
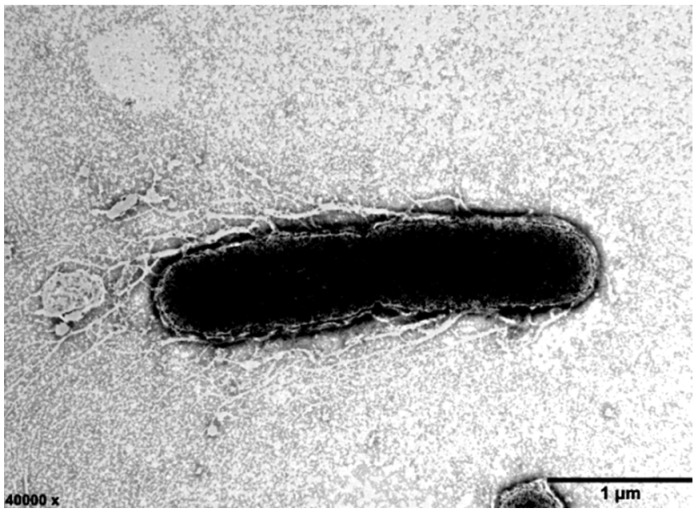
Transmission electron micrograph of the cell of strain ABI-127-1 grown on MA for 48 h. *Bar* 1 μm.

Strain ABI-127-1 was observed to grow well on MA, R_2_A, nutrient agar (NA), and trypticase soy agar (TSA), and the optimum medium was MA. Colonies of this strain were faint yellow, smooth, and circular when they were grown on MA. Cells of variable size (0.8–1.1 × 2.1–2.9 µm) were observed (Figure 1). Cells were rod-shaped, Gram-stain negative, aerobic, oxidase and catalase positive, and non-motile. Bacterial growth occurred at pH 5.0–10.0 (optimum, 7.0–8.0) and 15–35 °C (optimum, 28–30 °C) with 1–10% (*w*/*v*) NaCl (optimum, 2.5%) in MA. Differential physiological and biochemical characteristics between strain ABI-127-1 and close relatives in the genus *Qipengyuania* were shown in Table 1. Some unique properties such as the utilization of glucose, fructose, and lactate can be used to distinguish strain ABI-127-1 from close relatives. Also, the new isolate strain ABI-127-1 has been deposited in two counties, with certificate deposition numbers of CCTCC AB 2022122 (China Center for Type Culture Collection, CCTCC in China) or KCTC 92508 (Korean Collection for Type Cultures, KCTC in Republic of Korea), respectively.

### 2.2. Phylogenetic Characteristics of Strain ABI-127-1

**Figure 2:**
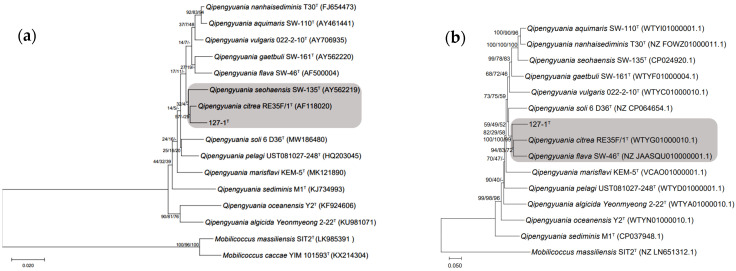
The constructed phylogenetic trees based on bacterial 16S rRNA gene sequences (**a**), and the multilocus sequence analysis (MLSA) using three bacterial housekeeping genes (*dna*A, *rop*A, and *phe*S) (**b**). Bootstrap values (≥50%) based on 1000 replications are shown at the branch points. *Mobicoccus masssiliensis* SIT2^T^ was used as the outgroup for both trees.

The almost complete 16S rRNA gene sequence (1474 bp) of strain ABI-127-1 was obtained and submitted to GenBank with the accession number of ON276358. It was found that it is identical to the gene sequence from the genomic sequence. The similarity comparison of 16S rRNA gene sequences showed the highest similarity values to *Q. citrea* RE35F/1^T^ (98.6%), *Q. aquimaris* SW-110^T^ (98.3%) and *Q. nanhaisediminis* CGMCC 1.7715^T^ (97.8%), respectively. The constructed phylogenetic tree based on bacterial 16S rRNA gene sequences indicated that strain ABI-127-1 formed a distinct separate phylogenetic line and clustered with type strain *Q. citrea* RE35F/1^T^ (Figure 2a). Additionally, the MLSA phylogenetic analysis based on three housekeeping genes (*dna*A, *rop*A, and *phe*S) also showed that strain ABI-127-1 occupied a separate phylogenetic branch and clustered with two strains of *Q. flava* and *Q. citrea* types (Figure 2b).

### 2.3. Phylogenomic Inference

**Figure 3:**
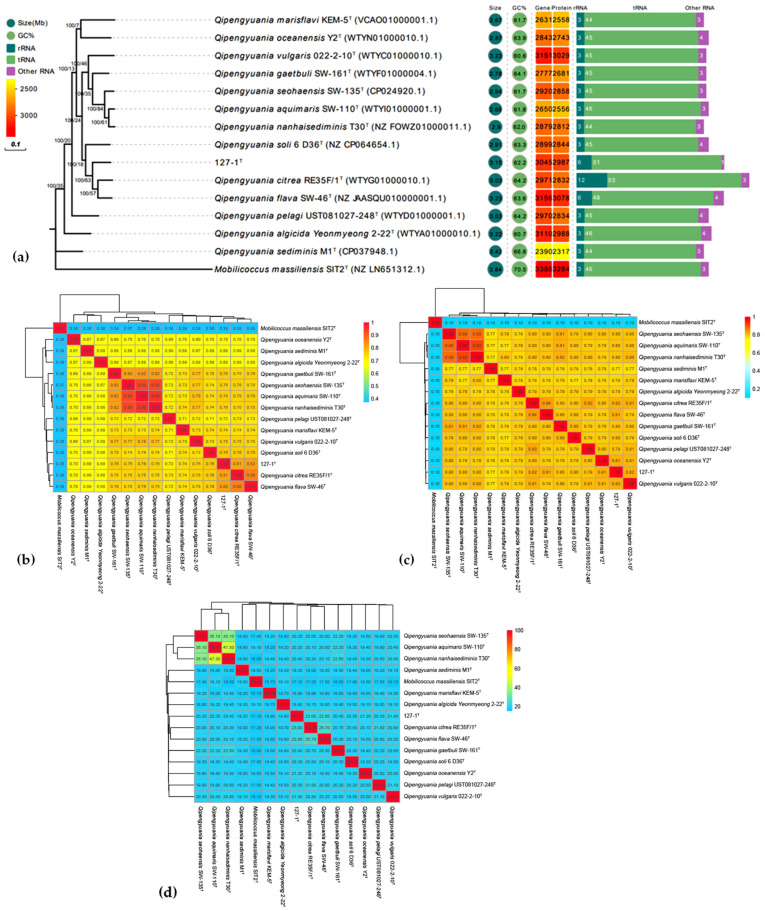
Phylogenomic tree (**a**) of strain ABI-127-1 constructed based on the up-to-date bacterial core gene (UBCG) method. Five genomic characteristics including the genomic size, DNA G+C content (%), numbers of protein-coding genes (CDs), gene numbers of rRNA and tRNA, and phylogenomic trees based on three phylogenomic similarity indexes were shown, including the ANI (**b**), AAI (**c**) and dDDH (**d**) value calculations.

The length of the draft genome sequence of strain ABI-127-1 (GenBank accession number JALJCR000000000) was approximately 3.15 Mb. The genomic DNA G+C content was calculated to be 62.15 mol%. To further infer the phylogenetic relationship between strain ABI-127-1 and closely related type strains with available genome sequences, the phylogenomic analysis was performed using an up-to-date bacterial core gene set (UBCG) [27,28,29]. As shown in Figure 3a, strain ABI-127-1 formed a separate branch in the constructed phylogenetic tree and clustered with *Q. citrea* RE35F/1^T^ and *Q. flava* SW-46^T^. This finding was consistent with the result obtained using the MLSA analysis (Figure 2b). 

Additionally, in order to fully resolve the taxonomic status of this new isolate, three measures of phylogenomic similarity including the average nucleotide identity (ANI), average amino acid identity (AAI), and digital DNA–DNA hybridization (dDDH) were performed. The calculated ANI, AAI, and dDDH values between strain ABI-127-1 and close relatives in the genus *Qipengyuania* were 77.1–81.2%, 64.1–75.6%, and 18.5–22.2%, respectively (Figure 3). Three phylogenomic similarities were far below the threshold values for novel species delineation [35]. Therefore, it is proposed that strain ABI-127-1 represents a potential novel species of the genus *Qipengyuania*.

### 2.4. Genomic Sequencing and Annotation of Strain ABI-127-1

**Figure 4:**
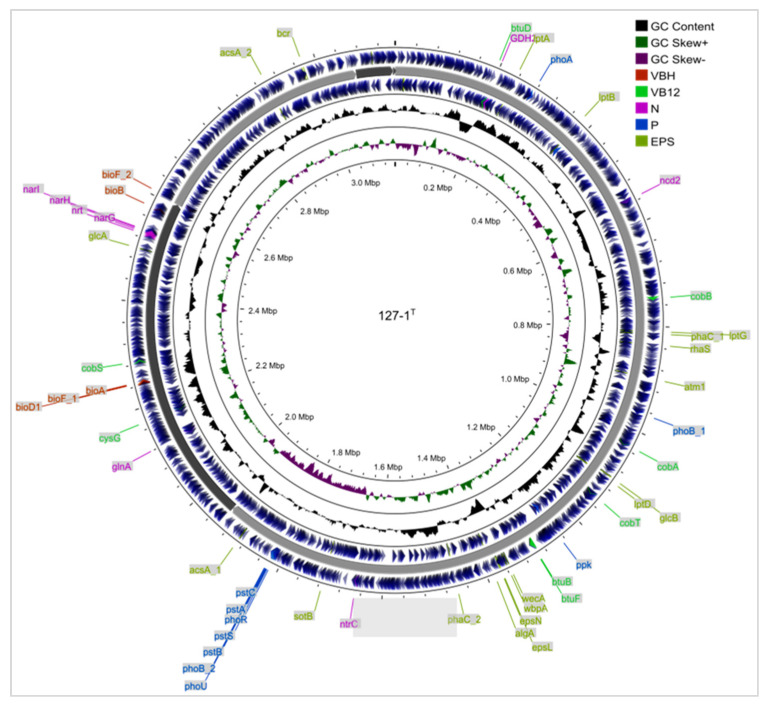
Circular representation of the genome of strain ABI-127-1. The scale of genome size is shown in the outer line. From the outer to inner circle: two outer circles showing protein-coding sequences (CDs) with different colors showing the genes in varied COG categories; the third circle shows the rRNA and tRNA genes (red); the fourth and inner fifth circles show DNA G+C content and G+C skew, respectively. Predicted biosynthetic gene clusters (BGCs) including biotin synthesis (VBH), vitamin B_12_ synthesis and transport (VB_12_), nitrogen (N) and phosphorus (P) metabolism, and EPS synthesis- and transport-related genes are also marked in the genome structure.

The obtained draft genome of strain ABI-127-1 consisted of six contigs with a genome size of 3,151,676 bp and an *N*_50_ value of 1,937,448 bp with an over 360× genome coverage. A circular representation of the draft genome is shown in Figure 4. Based on the genome annotation, the total number of predicted genes was 3089, and 3068 were identified as protein-coding genes. It also had 57 RNA genes including 51 tRNA and 6 rRNA genes. A total of 2215 genes (73.7%) were assigned to 26 functional categories based on the COG database, and 850 genes participated in the pathway of metabolism, which was classified as the primary function of the chromosome. Additionally, a total of 217 genes were not assigned to any known function, accounting for 9.8% of the total genes. KEGG analysis revealed that a total of 1550 proteins were classified into six pathways, and 42.8% were involved in different metabolism categories. GO analysis assigned total 2177 proteins into three functional groups, and the top five subgroups were assigned to the molecular function, biological process, cellular process, catalytic activity, and metabolic process, respectively.

Due to the close phylogenomic relationship between strains strain ABI-127-1 and *Q*. *flava* SW-46^T^ and *Q*. *citrea* RE35F/1^T^, the genomes of the three type strains were compared. For *Q*. *citrea* RE35F/1^T^, the genomic length was calculated as 3,031,321 bp consisting of 2971 predicted coding genes with a DNA G+C content of 64.2 mol%, whereas the genome size of strain *Q. flava* SW-46^T^ was 3,229,668 bp in which the DNA G+C contents of both strains were 63.6 mol% and 3156 predicted coding genes. The DNA G+C contents of three type strains all fell within the range of 60.5–66.8 mol% for members of the genus *Qipengyuania* [16,17,18,19]. 

### 2.5. Biosynthetic Gene Prediction for Active Metabolites

**Figure 5:**
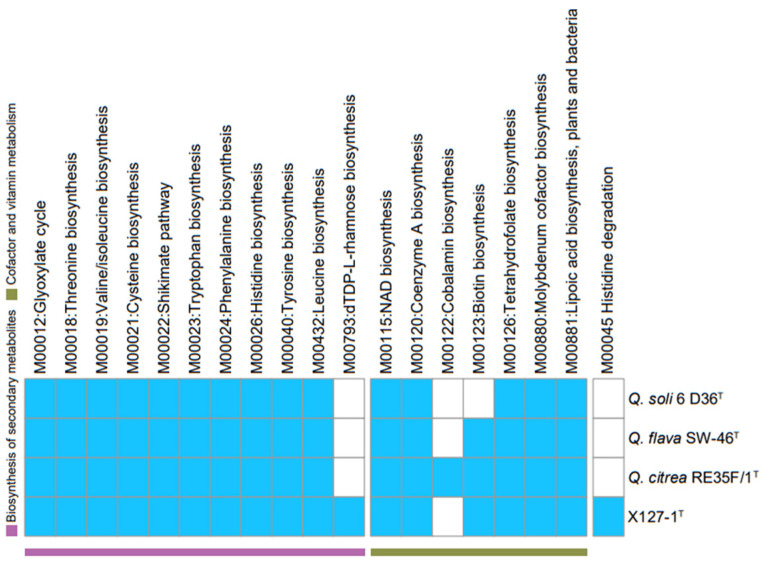
Comparison of the biosynthetic genes responsible for secondary metabolites, co-factors, and vitamin metabolisms between strain ABI-127-1 and three phylogenetic neighbors, *Q. citrea*, *Q. soli*, and *Q. flava* in the genus *Qipengyuania*.

Based on genomic annotation, a bioinformatics prediction of the biosynthetic genes in strain ABI-127-1 was performed. It was found that strain ABI-127-1 had genes which were responsible for biosynthetic modules for various co-factors and biotin B_12_ (Figure 4 and Figure 5), which are essential vitamins for algal growth and proliferation [33,34,36]. It indicated that strain ABI-127-1 owned the genetic basis to synthesize some interactive metabolites and exchanged them with the algal hosts during algae–bacteria interactions. In addition, two core genes (*eps*L and *eps*N) which were responsible for bacterial EPS biosynthesis were also found [37,38]. EPSs are considered to be the fundamental intermediates during algae–bacteria interactions thar occur within microscale phycosphere niches [10,39,40,41]. 

It is also noteworthy that the biosynthetic genes for the nucleotide sugar precursor dTDP-L-rhamnose were only found in strain ABI-127-1 (Figure 5) when compared with three phylogenetic neighbors including *Q. citrea* (from seawater), *Q. soli* (from mangrove soil), and *Q. flava* (from seawater). These genes are responsible for the production of structurally similar rhamnose polysaccharides in the bacterial cell wall, which is critical for maintaining cell shape, bacterial physiology, and virulence, and also for host interactions as vital chemical intermediates [35,42]. This finding may indicate the unique characteristic of the phycosphere niche that strain ABI-127-1 inhabits [39]. Also, the cobalamin biosynthesis pathway, the process by which bacteria and archea make cobalamin vitamin B12, was only found for strain *Q. citrea* isolated from the marine environment.

### 2.6. Growth-Promoting Potential of Strain ABI-127-1 towards Algal Host

**Figure 6:**
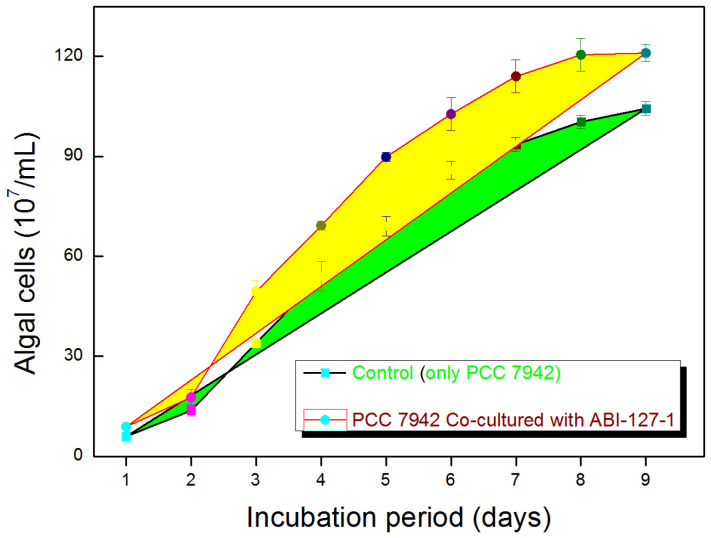
The microalgae growth-promoting potential of strain ABI-127-1 on PCC 7942 cells.

Our previous studies showed that some species in the phycosphere bacteria community demonstrated growth-promoting potential towards algal hosts [10,12,28,34]. Based on microalgae growth-promoting (MGP) bioactivity assaying, the total numbers of algal cells of PCC 7942 significantly increased when co-inoculated with strain ABI-127-1 with at least 16.5 ± 3.8% promotion efficiency (Figure 6). This indicates that the strain ABI-127-1 has obvious MGP activity. This finding may indicate that the possible association between strain ABI-127-1 and algal host, in spite of the nature of the detailed mechanism, remains unsolved. The current ongoing exploration of the potential associations in the co-culture circumstances based on multi-omics analysis is believed to offer insights into the mechanism, governing the interactions between strain ABI-127-1 and algal host PCC 7942 [6,7,8,43,44,45].

### 2.7. Culture Optimization for Bacterial EPS Production by Strain ABI-127-1

**Figure 7:**
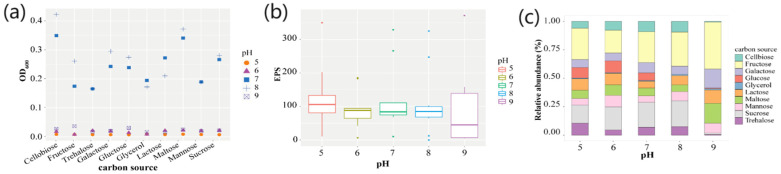
Culture optimization of bacterial growth and EPS production of strain ABI-127-1. The effects of the different pH and carbon sources on bacterial growth recorded at OD_600nm_ (**a**) and relative EPS production (**b**,**c**).

A preliminary investigation showed that two factors, the pH and carbon source in the media, were the main factors influencing the EPS accumulation in strain ABI-127-1. In this study, ten selected carbon sources including the cellbiose, fructose, galactose, glucose, glycerol, lactose, maltose, mannose, sucrose, and trehalose, and a pH ranging from 5.0 to 9.0, were selected and used for culture optimization for bacterial ESP production (Figure 7a). For the pH test, it was found that strain ABI-127-1 grew better at pH values ranging from 7.0 to 9.0. Moreover, it achieved the fastest growth rate when the cellobiose was used as the single carbon source when cultured at pH 8.0 (Figure 7b). However, according to the EPS accumulation measurements, the higher EPS yield was achieved when strain ABI-127-1 was cultured at pH 9.0 (Figure 7b). Thus, under the optimized conditions, the highest EPS yield of 118.2 ± 15.8 μg/mL (means ± SD) was obtained when cellobiose (10 g/L) was used as the carbon source and cultured at 30 °C at pH 9.0 (Figure 7c).

### 2.8. Bioflocculant Bioactivity of EPS Produced by Strain ABI-127-1

**Figure 8:**
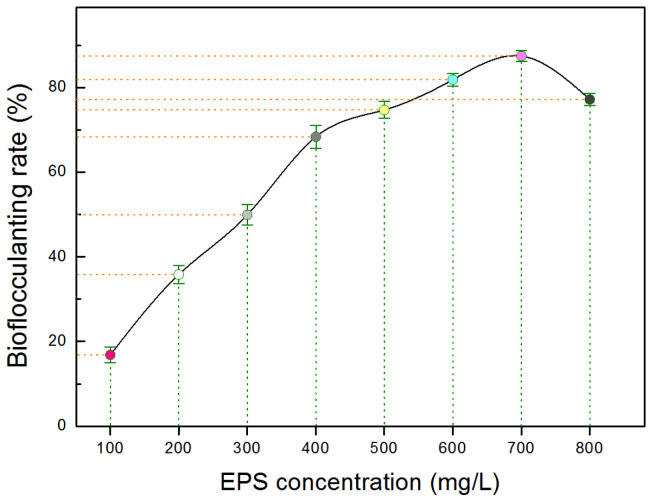
Bioflocculant activity of the bacterial EPS produced by strain ABI-127-1. The bioflocculant efficiency expressed with the relative bioflocculant rate (%).

To measure the production capacity of bacterial bioflocculants, the extracted EPSs produced by strain ABI-127-1 were subjected to bioflocculant activity evaluation [10,33,43]. A comparison of the bioflocculant effect under a series of EPS concentrations was performed. As shown in Figure 8. it can be seen that the bioflocculant efficiency of EPS produced by strain ABI-127-1 showed a concentration-dependent manner, and it reached a maximum of 86.4 ± 3.9% (means ± SD) when the EPS concentration was set as 0.70 g/L. It exhibited a higher bioflocculant capacity compared with other marine bacterial strains which were also isolated from the phycosphere microenvironments as previously reported [10,33,44]. The finding indicated that strain ABI-127-1 may serve as a new bacterial candidate with natural potential for the production of promising microbial bioflocculants derived from marine phycosphere niches. Furthermore, the ongoing chemical elucidation of the produced bacterial EPSs is believed to uncover the chemical nature of the natural bioflocculant instinct contributed by strain ABI-127-1. 

### 2.9. Bacterial Enchantment of Strain ABI-127-1 for Algal CO_2_ Fixation Efficiency

**Figure 9:**
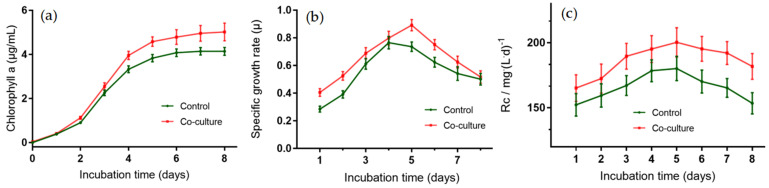
Effects of the co-cultivated strain ABI-127-1 on algal growth based on the measurements of chlorophyll *a* production (**a**), the growth rate (**b**), and also the CO_2_ fixation efficiency (**c**) of the PCC 7942 cells.

In a co-culture system, the cells of PCC 7942 were cultivated with strain ABI-127-1 in the optimized medium and aerated with 30 mL/min of 15% CO_2_ to determine the biomass productivity and CO_2_ fixation efficiency. As shown in Figure 9a, based on the chlorophyll *a* measurements, strain ABI-127-1 showed an obvious promoting effect on the microalgae biomass productivity with at least 20.8 ± 5.4% promotion efficiency. Chlorophyll *a* production reached the maximum value (17.6 ± 0.8%) by the sixth day. Figure 9b showed that, under co-culture circumstances, the peak growth rate of the PCC 7942 cells was increased to 0.892 g/L·day (0.715 g/L·day with the sole PCC 7942). Moreover, the time of peak growth rate was observed to be delayed from day 4 to day 5. The CO_2_ fixation efficiency by PCC 7942 cells when co-cultured with strain ABI-127-1 was increased from 2.25 ± 0.24% to 4.36 ± 0.39% within the first 2 days with increasing values from 152.3 ± 15.8 to 165.2 ± 17.3 mg/d, and then stabilized to over 10% within the following six days, and the average CO_2_ fixation efficiency under co-culture circumstance was 46.8 ± 5.6% during the whole experiment period (Figure 9c). This clearly shows the enhancing effect of the strain ABI-127-1 on algal CO_2_ fixation efficiency.

## 3. Materials and Methods

### 3.1. Bacterial Isolation and Culture Conditions

The model cyanobacterium *Synechococcus elongatus* PCC 7942 was purchased from the Freshwater Algae Culture Collection at the Institute of Hydrobiology (FACHB, Wuhan, China). For algal cultivation, PCC 7942 cells were grown in BG11 medium shaken at 150 rpm and at 30 °C under constant white-light illumination (30 μmol·m^2−1^·s^−1^) [31]. Bacterial strains were isolated from PCC 7942 cells using the dilution-to-extinction method as previously reported [33]. The isolation procedure was performed by spreading algal cells on marine 2216 agar (MA, BD, Franklin Lakes, NJ, USA) supplemented with an algal extract of PCC 7942 cells at a final concentration of 0.023 mg/L [35]. The isolated bacteria were routinely cultured on marine 2216 agar and preserved as 20 % (*v*/*v*) glycerol suspensions and stored at −80 °C.

For the comparative analyses, five type reference bacterial strains were purchased and used, including *Qipengyuania citrea* RE35F/1^T^ (=DSM 14432^T^) and *Qipengyuania seohaensis* SW-135^T^ (=DSM 16221^T^) obtained from the Deutsche Sammlung von Mikroorganismen und Zellkulturen GmbH (DSMZ, Braunschweig, Germany), *Qipengyuania nanhaisediminis* T30^T^ (=CGMCC 1.7715^T^) obtained from the China General Microbiological Culture Collection Center (CGMCC, Beijing, China), *Qipengyuania aquimaris* SW-110^T^ (=KCCM 41818^T^) obtained from the Korean Culture Center of Microorganisms (KCCM, Seoul, South Korea), and *Qipengyuania pelagi* UST081027-248^T^ (=NRRL 59511^T^) obtained from the Agricultural Research Service Culture Collection (NRRL, Peoria, IL, USA), respectively.

### 3.2. Phenotypic and Physiological Characterization

For the comparative analyses, all biochemical and physiological tests were performed where ABI-127-1 and five reference strains were routinely cultivated on MA at 30 °C following different experimental requirements. Bacterial growth was investigated on R_2_A agar (BD), trypticase soy agar (BD), MacConkey (BD), nutrient agar (BD), and marine agar (BD) after incubation at 30 °C for 2 days [43]. Colony color and morphology were observed. Morphological and cultural characteristics were investigated with cells grown on MA. Cell morphology and motility were observed using light microscopy (BH-2; Olympus, Tokyo, Japan) and transmission electron microscopy (JEM-1200; JEOL, Tokyo, Japan), respectively, after bacterial incubation at 30 °C for 48 h. The gram reaction was tested using the bioMérieux Gram 2 Kit (bioMérieux, Marcy-l’Étoile, France) according to the manufacturer’s instructions. Oxidase activity was determined with a 1% (*w*/*v*) solution of tetramethyl-*p*-phenylenediamine. Catalase activity was tested with a 3% (*w*/*v*) H_2_O_2_ solution [37]. Growth at different temperatures (4, 10, 20, 25, 30, 37, 40, 45, and 50 °C) and pH values (4.0–11.0, at intervals of 0.5 pH units) was determined after culture in MB at 30 °C for up to 10 days [38]. Growth at various NaCl concentrations (0–15.0%, *w*/*v*, with the interval of 0.5%) was investigated in MB and cultured in a rotary shaker for 7 days.

### 3.3. Phylogenetic Analysis

For the phylogenetic analysis, the genomic DNA extraction, PCR amplification of the bacterial 16S rRNA gene using the universal primers of 27F/1492R, and the sequencing of the PCR products were performed as described previously [46,47]. The identification of the phylogenetic neighbors and calculation of the 16S rRNA gene sequence similarities were performed using the sequences of the type strains available from GenBank and Ez-Taxon using the BLAST program and online Ez-Taxon database (https://www.ezbiocloud.net, accessed on 26 June 2023). The 16S rRNA gene sequences were aligned using the Clustal_W program, and the phylogenetic tree was constructed via the neighbor-joining (NJ), maximum likelihood (ML), and maximum parsimony (MP) algorithms implemented in the MEGA software (version 7.0) using the default settings [48]. Bootstrap confidence analysis was carried out with 1000 replicates for evaluating the robustness of the tree topologies. 

### 3.4. Genome Sequencing, Assembly, and Annotation

Strain ABI-127-1 was cultured in marine 2216 (MB) (Difco) at 30 °C and the cells were harvested in the mid-logarithmic phase. The genome DNA was extracted and purified using the QIAamp DNA Mini Kit (Qiagen, Hilden, Germany), according to the manufacturer’s instructions, and sequenced by the PacBio RS II Single Molecule Real Time (SMRT) sequencing platform (Pacific Biosciences, Menlo Park, CA, USA) as described previously [10]. For PacBio sequencing, the genomic DNA was sheared to 10 kb using g-TUBE kit (Covaris, Woburn, MA, USA) and converted into the proprietary SMRTbell^TM^ library format using the PacBio RS DNA Template Preparation Kit. The constructed library was then sequenced on the SMRT platform. A total of 1078 Mb filtered high-quality reads with average 360-fold genome coverage were assembled into one contig. The constructed contig was then connected into one chromosome using SOAPdenovo software (version 2.0) [49]. Based on the genome sequence of strain ABI-127-1, the protein-coding regions were predicted by Glimmer (Version 3.02), and all the gene functions were annotated by aligning to the GO (https://geneontology.org/, accessed on 10 July 2023), COG (https://www.ncbi.nlm.nih.gov/COG, accessed on 10 July 2023), and KEGG (http://www.kegg.jp, accessed on 12 July 2023) database, respectively. 

### 3.5. Phylogenomic Calculations

The DNA G+C content was calculated based on the obtained genome of strain ABI-127-1. Three phylogenomic measures of similarity including the average nucleotide identity (ANI), average amino acid identity (AAI), and digital DNA–DNA hybridization (dDDH) [50] were used. The values were calculated using an online EzAAI tool (http://leb.snu.ac.kr/ezaai, accessed on 7 July 2023), and GGDC tool (http://ggdc.dsmz.de/distcalc2.php, accessed on 10 July 2023) using the default parameters [51], respectively. The genome-based phylogenetic tree was constructed using an up-to-date bacterial core gene set (UBCG) consisting of the bacterial core of 92 genes as described previously [52].

### 3.6. Multilocus Sequence Analysis (MLSA)

For the multilocus sequence analysis (MLSA), three bacterial housekeeping genes including *dna*A, *rop*A, and *phe*S were used [53]. A total of 13 genome sequences of the closely related reference type strains in the genus *Qipengyuania* were retrieved from the GenBank database (https://www.ncbi.nlm.nih.gov/genome, accessed on 10 July 2023). Three housekeeping genes were extracted from the genome sequence using PhyloSuite software (version 1.2.2) [54]. Phylogenetic trees were reconstructed by MEGA_X (version 4.0) using three algorithms and using bootstrap analysis based on 1000 replications.

### 3.7. Bacterial Growth and EPS Production Measurements

For the culture optimization procedure, 1 mL of 24 h fresh bacterial culture of strain ABI-127-1 was taken and mixed with 24 mL of fresh 2216 medium. Then, a 150 μL of the mixture was added into the wells of the 96-well microplate containing different carbon sources and cultured with shaking at 60 rpm/min for 72 h [55]. During the culture period, the bacterial growth rate was monitored with the measurement of the optical density change recorded at OD_600 nm_ every three hours. Ten carbon sources including cellbiose, fructose, galactose, glucose, glycerol, lactose, maltose, mannose, sucrose, and trehalose, which were added with the final concentration of 10 g/L and a pH range of 5.0–9.0 with 1.0 as the interval, were used for the measurements [56]. The extraction and the quantification analysis of the produced EPS were performed using the phenol sulfuric acid method, as reported previously [57]. 

### 3.8. Measurements of Bioflocculant and MGP Bioactivities

The evaluation of the bioflocculant activity of the EPS was performed according to procedures reported previously [10,33,58]. The prepared EPS substances were dissolved in the distilled water for a further bioactivity assay. Measurements using kaolin clay suspension flocculation assay calculated and expressed as bioflocculation rate (%) were used and were performed using 96-well microplates with at least six replicates [59]. The microalgae growth-promoting (MGP) activity of strain ABI-127-1 toward PCC 7942 cells in a co-culture system was evaluated as reported previously [60] and performed in a SpectraMax M2 model 96-well microplate reader (Molecular Devices, LLC, San Jose, CA, USA). All the obtained results were expressed as means ± SD. 

### 3.9. Measurement of Algal Carbon Fixation Efficiency in Co-Culture System

During the co-culture, the chlorophyll was extracted from microalgae cells via soaking in DMSO/acetone solution (1/2, *v*/*v*) and then measured as previously reported [61]. In total, 20 mL of the samples was centrifuged at 6000× *g* for 10 min and dried at 65 °C for 24 h to obtain the dried biomass weight. Biomass concentration (g/L) was calculated from the microalgae dry weight produced per liter [62]. The growth rate (GR, g/L·day) was calculated using the following formula, GR = (M1 − M2)/(t1 − t2), where M1 was the biomass concentration at the time of t1 and M2 was the biomass concentration at the time of t2. The carbon content of dry algae cells (*X_cbm_*, %) was analyzed using a CHN element analyzer (Vario Macro Cube, Elementar, Germany), as previously reported [63]. The algal CO_2_ fixation efficiency (mg/L·d) was calculated according to the following formula, *R_c_* = *P_x_·X_cbm_·M_CO_2__/M*, where the *R_c_* was for the CO_2_ fixation rate (g/L·d), Pxwas was for the growth rate (g/L·d), *X_cbm_* was for the carbon content of dry algae cells (%), *M_CO_2__* was for the molecular mass of CO_2_, and *M_c_* was for the molecular mass of carbon, respectively.

## 4. Conclusions

Based on a phylogenetic analysis using the bacterial 16S rRNA gene alignment, the yellow-pigmented bacterial strain ABI-127-1, which was isolated from the cultivable phycosphere microbiota of model cyanobacterium, *Synechococcus elongatus* PCC 7942, was shown to belong to the genus *Qipengyuania*. Additional phylogenomic analysis obtained the unequivocally separated strain ABI-127-1 from close relatives and further confirmed the phylogenetic position of this isolate to represent a potential novel species of this genus. This new isolate demonstrated obvious promoting potential towards both algal growth and CO_2_ fixation efficiency. Additionally, the bacterial production of active bioflocculant exopolysaccharides was also presented. The current study reveals the potential environmental and biotechnological implications of this new microalgae growth-promoting bacterium recovered from unique phycosphere microenvironment.

## Figures and Tables

**Table 1:** Characteristics differentiating strain ABI-127-1 from the phylogenetic neighbors within the genus *Qipengyuania*.

Characteristic	1	2	3	4	5	6
Isolation source	marine phycosphere	seawater	seawater	seawater	seawater	marine sediment
Colony color	Yellow	Yellow	Orange	Orange	Orange	Orange
Motility	−	−	−	−	−	+
Nitrate reduction	+	+	−	−	+	−
Tolerance to NaCl (%, *v*/*v*, optimal)	1.0–10.0 (2.5)	1.0–10.0 (3.0)	1.0–9.0 (2–3)	0.5–15.0 (3.0)	0.5–9.0 (2–3)	0–10.0 (3.5)
Growth temperature (°C, optimal)	15–35 (28–30)	4–37 (25–30)	10–40 (30–35)	10–41 (30–35)	12–40 (30–36)	10-40 (30–35)
Growth pH range (optimal)	5–10 (7–8)	5–10 (7–8)	5–10 (7–8)	5–10 (7–8)	5–10 (8–9)	5–10 (7–8)
Utilization of						
Glucose	−	+	+	+	+	+
Fructose	+	−	−	−	−	−
Lactate	+	−	−	−	−	−
16S rRNA gene similarity (%)	−	98.64	97.79	98.29	97.29	97.79
DNA G+C content (mol%)	62.15	62.01	62.18	62.17	60.40	59.51

Strains: 1, ABI-127-1; 2, *Q. citrea* RE35F/1^T^; 3, *Q. seohaensis* SW-135^T^; 4, *Q. aquimaris* SW-110^T^; 5, *Q. pelagi* UST081027-248^T^; 6, *Q. nanhaisediminis* T30^T^.

## Data Availability

All relevant data are included in the manuscript.
